# Hemorrhage from the Uterus Continually during Pregnancy

**Published:** 1861-03

**Authors:** P. M. Tidwell

**Affiliations:** Fairburn, Ga.


ATLANTA

Medical and Surgical Journal.

VOL. VI.] MARCH, 1861. [NO. 7.

### ORIGINAL COMMUNICATIONS.

### ARTICLE I.

*Hemorrhage from the Uterus Continually during pregnancy.* By P. M. Tidwell, M. D., of Fairburn, Ga.

About the first of February 1860, I was requested to prescribe for Mrs. C——, aged 43, and the mother of 13 children, who was reported, by her husband, to be in a very singular, and extremely unpleasant condition. From him the following history was obtained, before prescribing for his wife:

In October of the previous year, irregularity of menstruation was noticed. The periods which lasted near a week came on every three weeks instead of four. The interval gradually shortened to eight or ten days, and finally, at the time I was consulted, and for a month previously, the hemorrhage was constant. The usual prescription for uterine hemorrhage, lead and opium, was made, which I afterwards learned was not effectual in arresting the discharge, but from the failure to take the prescription promptly, could not ascertain to what extent the hemorrhage might be under the control of lead.

From the third day of February, I heard no more of the case, till the 27th of April, when I was again consulted in regard to her case, and visited her with the view of ascertaining more certainly her true condition. It appeared from the history given by the patient and her husband, that she had been attended for some time by a physician in the neighborhood—in whose absence I had been called—and that the remedies given by him had made no favorable impression. She had been impressed by her female friends, with the idea that she was passing the "critical period," and that time would be required for the system to recover from the derangement consequent upon this change. She had been able to attend to her domestic duties for most of the time till the first of March, notwithstanding the constant flow. Since that time the hemorrhage is so profuse, occasionally, that she is confined in bed. Distressing nausea, and not unfrequently profuse vomiting, added still more to the unpleasantness of her condition. She reported that some months previously, she had passed through regular "morning sickness," as in her pregnancies, and in addition, some three weeks previous to my visit had felt a movement which might be attributed to pregnancy. These facts had awakened in her the suspicion of a different state of things from that of the "change of life," as she had been persuaded by her friends. Examination per vaginum and manipulation over the abdomen soon gave me the means of verifying her supposition. No disease or difficulty of any kind was discovered about the os uteri, but sufficient evidences of development of the uterus to authorize the opinion of pregnancy of six months standing, and distinct movement of the fœtus observed. While I could not really doubt the existence of pregnancy, I did not conceal my astonishment of the uninterrupted development with such constant, and occasionally copious discharge of blood from the uterus. And while I confessed to her that it was the first case of the kind I had witnessed, informed her that such cases were not unknown to the profession, though not of frequent occurrence. In compliance with the request of the patient to give every attention to her case, promised to do so, but with the history of her management in the past in view, did not risk a promise of relief from the continuance of hemorrhage, certainly.

The following prescription was made for the present:

℞. Plumbi acetas, grs xii.
Opium, grs iv.
M. and make pills No. iv.

One to be taken every four hours.

*April 28th.*—Saw patient this morning, and found that the pills had produced so much nausea that it was found advisable to suspend their use, after two had been taken. I then prescribed the anti-hemorrhagic preparation of Prof. Meigs, composed of nutmeg and alum, and the following injection:

℞. Plumbi acetas, ʒ ss.

Zinci Sulph, grs xx.

Tinct catechu,

Tinct opii, aa ʒ ii,

Aqua Rosæ, ℥ vi.

M. and inject into vagina twice a day.

At the same time advised the use of extract of butternut and hyoscyamus in sufficient quantities to keep the bowels laxative.

*April 30th.*—The prepartion of alum had also produced unpleasant effect upon the stomach and could not be continued. No improvement to this time having resulted from any of the means used, the following was advised:

℞. Acid, sulph. aromat., gtts xxx.
Syrup gentian, ℥ vi.
M. and give two fluid ounces every four hours.

Extract butternut continued as before.

*May 4th.*—The hemorrhage, though much lessened in quantity, still continues—no unpleasant effect from the acid and gentian—same prescription continued.

*May 9th.*—Patient about the same—continued, though moderate hemorrhage—not profuse since the use of the acid was commenced—the same treatment continued.

*May 13th.*—Patient since last visit has suffered with the return of nausea and vomiting, hemorrhage has also increased being now quite profuse—suffers with pain in the back and hips—strength greatly reduced. Patient now much alarmed for her safety, and suggests the propriety of releaving her from the cause of her distress and danger, by premature dilivery. She fears the strength of her system will not allow her to hold up under the constant drain for the two or three months which are yet wanting to complete the period of gestation. In the request, the husband joins. To this I objected, assuring them that no very urgent necessity existed for such procedure, and that should such exigency be manifest, prompt measures should be instituted. Persisting, however, in their united petitions, I proposed a compromise by asking consultation with other physicians, and promising to carry out the plan determined upon. To this their ascent being given, I ordered the acid and syrup of gentian as before, and left to make arrangement for consultation the next day.

*May* 14*th*.—Patient continues as on yesterday—no return of nausea. After thorough investigation and free consultation with the physician selected, it was determined that no necessity existed for the expulsion of the fœtus. Prescribed anodyne of opium in addition to the acid and syrup.

15*th*.—No material change—various astringents internally and in the form of wash were resorted to, but owing to the irritable condition of the stomach rarely tolerated, and the local applications were not particularly servicable. Anodynes of the opiate preparations, and rest were the only course that promised any benefit, and were instituted as the means to enable her to go through the full period safely.

About the 20th of May the case was placed in the hands of another physician and I know no more of the particulars of the treatment. I learned, however, that it resulted in premature delivery, without being artificially produced, during the seventh month of pregnancy; and that up to the time of delivery from conception, as well as that period could be determined, not a day, nor perhaps an hour, passed without uterine hemorrhage. The lady is said to be now in the enjoyment of fine health.

